# My bad tooth and why it matters: the neglected value of "Medical Touch"

**DOI:** 10.5116/ijme.6a5f.3a9d

**Published:** 2026-08-13

**Authors:** Ami Schattner

**Affiliations:** 1 The Faculty of Medicine, Hebrew University and Hadassah Medical School, Jerusalem, Israel

**Keywords:** Physical examination, clinical training years at medical school, dental physical examination, patient reassurance

## To the Editor

It all started with a common, nagging toothache, and being too busy to schedule an appointment with my dentist in town, I went to the dental school on the opposite side of the road from the hospital. Alon saw me there. He appeared to be a young faculty member, and our first meeting remains embedded in my memory, though years have passed. I don't have much love for dentists and their treatments. But I can still feel his gloved fingers probing every corner of my mouth, gently and thoroughly. His physical examination took just a few minutes, but it gave me such a good feeling of expertise, seriousness and devotion that never faded since. My problem was solved soon enough, but when I needed more complex dental reconstruction, I came back straight to Alon and, over an extended series of visits, received the same top-notch care to my never-ending gratitude.

Nowadays, when my students are in their internal medicine rotation, they question the need to palpate, percuss and auscultate in the era of point-of-care ultrasound and echocardiography. When screens demand more and more of their time and attention at the expense of patient face-time and patient care. And CT scans sometimes precede communication and examination - I am reminded of my first meeting with Alon, and how, in a different yet similar discipline, a high-quality physical examination produced an everlasting impression, trust, and bonding.

The declining skills in the teaching and performance of the physical examination by students and residents is hardly surprising,^1^ in sharp contrast to its essential and enduring value.^2^^,^^3^ As a reminder, the physical examination has a proven power, together with the history and basic tests, to diagnose approximately 75% of admitted patients with notable low cost, safety, unqualified availability, and power to yield important negative findings which are essential to clinical reasoning.^1^^,^^4^ Since modern clinical reasoning emphasizes a hypothesis-driven approach, physical examination too had better be problem-oriented, rather than a comprehensive head-to-toe assessment. As such, it does not take long to perform, enhancing the value (of information gained) per time (invested) ratio. Unfortunately, there are multiple barriers to the implementation of physical examination skills in medical graduates. For example, time constraints and over-reliance on technology-based diagnosis; shortage of role models with expertise and self-confidence in performing and teaching examination and interpreting findings; the distancing from the bedside; and the need to deal with a continuous influx of information, creating constant cognitive overload. The Stanford 25 initiative of disseminating essential finite examination skills, together with additional promising strategies 1 may, if widely incorporated by educators, stop the deterioration and improve widespread current deficiencies.^2^^,^^5^

In addition, as my personal experience with Alon taught me, the physical examination in itself has important healing effects the “medical touch”.^6^ Both patients and physicians report that the ritual of the examination and the intimacy involved have an important added value, transmitting reassurance and empathy, establishing roles, conveying expertise, and establishing patient satisfaction, trust, and relationship. This concept was well-supported by an impressive systematic review from Stanford covering the literature over 20 years and seeking to identify practices (evidence and narrative-based) that foster clinician presence and connection with the patient in clinical encounters.^7^ Among selected nonverbal communication, “use of expressive touch” stands out, later to be among 13 practices of value, under the heading “Recognize the power of touch”. Even though it was not finally included (notably,patients rated it higher than clinicians), touch in the encounter, and especially during examination, carries an inherent value and may leave a lasting impression.

Much more than a positive caring experience for the patient (and a wealth of information for the clinician), this physical contact is associated with lasting adherence, healing, and improved health outcomes.^6^^, ^^8^^-^^10^ For example, six experienced physician-educators from the University of Calgary reflected on their experiences of touch in their practice and teaching.^8^ The main theme identified was their use of touch as an important tool of communication, conveying to the patients “presence”, shared humanity and empathy. Buono and colleagues identified 36 empirical studies focusing on touch in health care.^9^ While stressing touch-mediated communication and its value in patient reassurance, signaling empathy and achieving “strong affective bonds” and mutual attunement, they also discuss the need for understanding boundaries and developing methodology for future research. Finally, the two studies that focus on the patients’ perceptions and experience of being touched by their general practitioner make the distinction between task-oriented touch (while a task is being performed) and expressive, spontaneous touch.^6^^,^^11^Both are welcomed by patients, seen as an additional tool to improve communication.^11^ The physical examination, in particular, is perceived as an important ritual wherein the doctor’s role and skills and caring are established.^6^ These concepts are as valid during the clinical training years at medical school, though it remains a relatively neglected area.^12^

Thus, rather than being ancient history, the dental physical examination by Alon is still vivid in my mind, encouraging me as I demonstrate to my students why and how physical examination and touch in health care are still pivotal.

### Conflicts of Interest

The author declares that there is no conflict of interest.
